# MHC class II transactivator CIITA induces cell resistance to Ebola virus and SARS-like coronaviruses

**DOI:** 10.1126/science.abb3753

**Published:** 2020-08-27

**Authors:** Anna Bruchez, Ky Sha, Joshua Johnson, Li Chen, Caroline Stefani, Hannah McConnell, Lea Gaucherand, Rachel Prins, Kenneth A. Matreyek, Adam J. Hume, Elke Mühlberger, Emmett V. Schmidt, Gene G. Olinger, Lynda M. Stuart, Adam Lacy-Hulbert

**Affiliations:** 1 Benaroya Research Institute, Seattle, WA 98101, USA.; 2 National Institute of Allergy and Infectious Diseases (NIAID) Integrated Research Facility, Frederick, MD 21702, USA.; 3 Massachusetts General Hospital, Boston, MA 02114, USA.; 4 Department of Genome Sciences, University of Washington, Seattle, WA 98109, USA.; 5 Boston University School of Medicine, Boston, MA 02118, USA.; 6 National Emerging Infectious Diseases Laboratories, Boston University, Boston, MA 02118, USA.; 7 Merck and Co., Inc, Kenilworth, NJ 07033, USA.; 8 MRIGlobal, Gaithersburg, MD 20878, USA.; 9 Bill and Melinda Gates Foundation, Seattle, WA 98109, USA.; 10 Department of Immunology, University of Washington, Seattle, WA 98109, USA.

## Abstract

Recent outbreaks of Ebola virus (EBOV) and severe acute respiratory syndrome coronavirus 2 (SARS-CoV-2) have exposed our limited therapeutic options for such diseases and our poor understanding of the cellular mechanisms that block viral infections. Using a transposon-mediated gene-activation screen in human cells, we identify that the major histocompatibility complex (MHC) class II transactivator (CIITA) has antiviral activity against EBOV. CIITA induces resistance by activating expression of the p41 isoform of invariant chain CD74, which inhibits viral entry by blocking cathepsin-mediated processing of the Ebola glycoprotein. We further show that CD74 p41 can block the endosomal entry pathway of coronaviruses, including SARS-CoV-2. These data therefore implicate CIITA and CD74 in host defense against a range of viruses, and they identify an additional function of these proteins beyond their canonical roles in antigen presentation.

## The CIITAdel keeps viruses at bay

A better understanding of cellular mechanisms involved in viral resistance is needed for the next generation of antiviral therapies. Bruchez *et al.* used a transposon-mediated gene-activation screen to search for previously unreported host restriction factors for Ebola virus (see the Perspective by Wells and Coyne). The authors found that a transcription factor, major histocompatibility complex class II transactivator (CIITA), induces resistance in human cell lines by directing the expression of the p41 isoform of the invariant chain (CD74). CD74 p41 then disrupts cathepsin-mediated Ebola glycoprotein processing, which prevents viral fusion and entry. CD74 p41 can also stymie the endosomal entry of coronaviruses, including severe acute respiratory syndrome coronavirus 2 (SARS-CoV-2). This work should inform future treatments against cathepsin-dependent viruses such as filoviruses and coronaviruses. Additionally, the screening strategy used may serve as a blueprint for uncovering resistance mechanisms against other dangerous pathogens.

*Science*, this issue p. 241 see also p. 167

## Teaser

CIITA and CD74 are host antiviral factors that inhibit Ebola and SARS virus fusion and entry.

Recent and ongoing outbreaks of Ebola virus (EBOV) in Africa (*1*) and the severe acute respiratory syndrome coronavirus 2 (SARS-CoV-2) pandemic highlight the need to identify additional treatment strategies for viral infections, including approaches that might complement traditional antivirals. Of particular interest is the identification of host-directed therapies that target common vulnerabilities and may be efficacious against multiple viruses, including those that may emerge in the future.

**Fig. 1:**
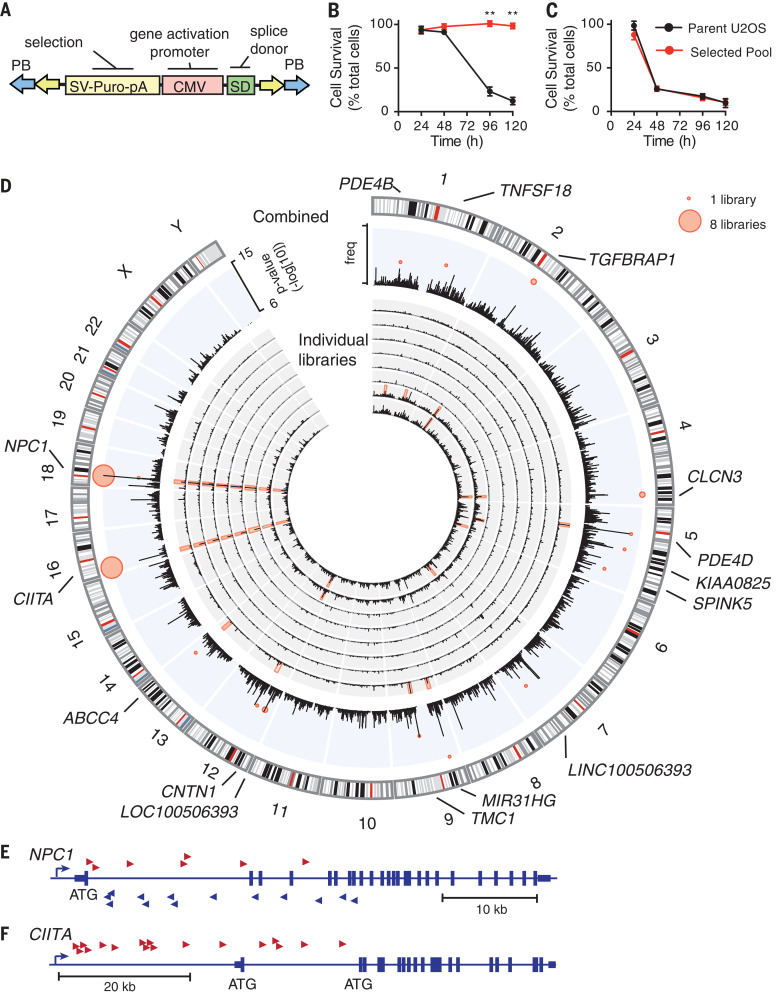
Transposon-mediated activation tagging generates mutant cells resistant to Ebola. (**A**) Modified PB transposon. SV-Puro-pA, puromycin selection cassette; CMV, cytomegalovirus promoter; SD, splice donor. (**B** and **C**) Resistance of selected cells to EboGP-VSV (B) and VSVg-VSV (C). Data are means ± SD of *n* = 3 replicates for one representative pool. Student’s *t* test; **P < 0.01. (**D**) Distribution of transposon insertions. Inner rings show insertions per 1 Mb for individual libraries (black histograms) and CISs (*P* < 10*^−^*^7^). Outer ring shows combined insertions for all libraries (black histogram) and lowest *P* value for CISs (red bubble plot). Point size represents the number of libraries with the CIS. freq, frequency. (**E** and **F**) Cumulative independent insertions from all eight libraries mapping to *NPC1* (E) and *CIITA* (F).

We set out to identify host pathways of cellular resistance to pathogens with pandemic potential, using a transposon-mutagenesis–forward genetic approach. We used a modified PiggyBac (PB) transposon (Fig. 1A), which stimulates or disrupts the expression of neighboring genes, thereby allowing an interrogation of both gene activation and inactivation in a single screen (*2*). Transposon-mutagenized libraries were treated with Ebola glycoprotein (EboGP)–expressing recombinant vesicular stomatitis virus (referred to as EboGP-VSV). Susceptible wild-type U2OS cells died after 3 to 4 days of treatment, whereas surviving cells could be expanded from mutagenized libraries and exhibited stable resistance to rechallenge with EboGP-VSV (Fig. 1B). These cells showed no cross-resistance to vesicular stomatitis virus (VSV) containing the VSV glycoprotein (VSVg-VSV) (Fig. 1C), which suggests that most of the resistance mechanisms selected in this screen targeted EboGP-mediated entry.

We identified candidate resistance genes by identifying genomic regions with high numbers of transposon insertions [referred to as common insertion sites (CISs)] (*3*). Combining data from eight independent screens revealed seven genomic loci with highly statistically significant (*P* < 10^−8^) CISs that occurred in more than one screen, representing high-confidence candidate-resistance mutations (Fig. 1D, outer ring). Likely target genes of transposon insertions were identified on the basis of transposon insertion position and orientation (Fig. 1D and table S1). We focused on the two genes that were found in all eight screens using the most stringent criteria.

The first of these was *NPC1*, located on chromosome 18. All transposon insertions at this site were intragenic in both sense and antisense orientations, and all were predicted to disrupt *NPC1* expression (Fig. 1E). This is consistent with the role of *NPC1* as the EBOV receptor (*4*, *5*) and validates our screening approach. Notably, U2OS cells are haploid at the *NPC1* locus (*6*), and these transposon insertions are therefore predicted to generate *NPC1-*null cells, which explains why *NPC1* was the only predicted gene-disruption mutant identified as a high-stringency candidate gene.

**Fig. 2:**
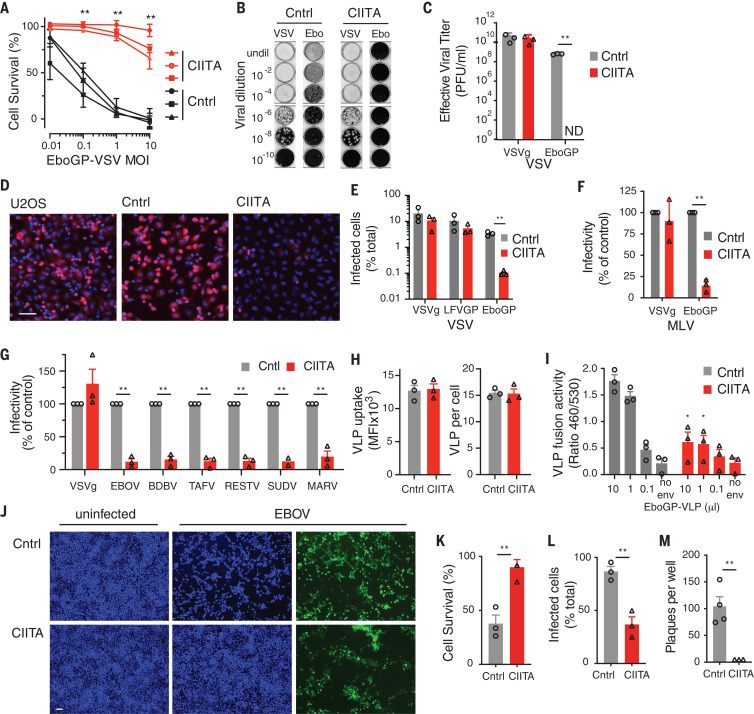
Identification of CIITA as an Ebola restriction factor. (**A**) Resistance of CIITA-overexpressing and control (Cntrl) U2OS cells EboGP-VSV. MOI, multiplicity of infection. (**B** and **C**) Plaque formation assays (B) and effective viral titer (C) for control and CIITA-overexpressing U2OS cells infected with VSVg-VSV (VSV) and EboGP-VSV (Ebo). undil, undiluted; PFU, plaque-forming units. (**D**) Representative images of CIITA-transfected (CIITA), control-transfected (Cntrl), and unmanipulated U2OS cells (U2OS) infected with mCherry-expressing EboGP-VSV (red) and stained with Hoechst 33342 to resolve cell nuclei (blue). (**E** to **G**) Infection of control and CIITA-expressing U2OS cells by recombinant VSV pseudotyped with EboGP, LFVGP (Lassa virus GP), or VSVg (E); single cycle murine leukemia virus (MLV) pseudotyped with VSVg and EboGP (F); or single cycle HIV pseudotyped with VSVg or GP from EBOV, Taï Forest virus (TAFV), Bundibugyo virus (BDBV), Sudan virus (SUDV), Reston virus (RESTV), or Marburg virus (MARV) (G). (**H** and **I**) Internalization (H) and fusion (I) of EboGP-VLPs by control and CIITA-overexpressing U2OS cells. No env, nonenveloped control VLPs. (**J** to **M**) Infection of control and CIITA-overexpressing U2OS cells by infectious EBOV measured by imaging of GFP reporter (green) and cell nuclei (blue) (J), cell survival (K), infected cells (L), or plaque formation (M). Data are means ± SEM of three independent experiments [(A) to (I)] or experiments with three independent cell clones [(K) to (M)]. Student’s *t* test [(A), (C), and (K) to (M)] or analysis of variance (ANOVA) with Tukey’s multiple comparison test [(E) to (I)]; *P < 0.05; **P < 0.01; ND, not detected. Scale bars, 100 μm.

All transposon insertions at the second CIS—located on chromosome 16—were upstream of the gene *CIITA* and were oriented in the sense orientation, consistent with activation of expression (Fig. 1F and fig. S1). CIITA overexpression in wild-type U2OS cells increased cell survival, reduced green fluorescent protein (GFP) reporter expression, and completely inhibited plaque formation, which confirms that CIITA increases resistance to EboGP-VSV 100- to 1000-fold (Fig. 2, A to E, and fig. S2). CIITA-overexpressing cells were also resistant to EboGP-pseudotyped single cycle viruses (Fig. 2, F and G), which strongly suggests that CIITA inhibits viral entry rather than targeting viral transactivators as suggested for HIV and human T cell leukemia virus (HTLV) (*7*, *8*). Furthermore, using EboGP virus–like particles (EboGP-VLPs) carrying β-lactamase (*9*), we found that CIITA did not affect the internalization of EboGP-VLPs into cells (Fig. 2H), but it blocked viral fusion, which occurs in the endosome (*10*) (Fig. 2I). CIITA-expressing U2OS cells were also highly resistant to infection by high titers of native EBOV, showing reduced reporter gene expression, cell death, and plaque formation (Fig. 2, J to M). CIITA expression did not inhibit replication of an EBOV minigenome, which indicates that CIITA does not act on the viral replication complex (fig. S3). Furthermore, CIITA inhibited infection mediated by glycoproteins (GPs) from a range of EBOV species—including Sudan, Zaire, and Reston—as well as by those from the distantly related filovirus Marburg virus (Fig. 2G). Thus, CIITA induces broad antiviral activity against EBOV and other pathogenic filoviruses through the inhibition of viral GP-mediated entry.

**Fig. 3:**
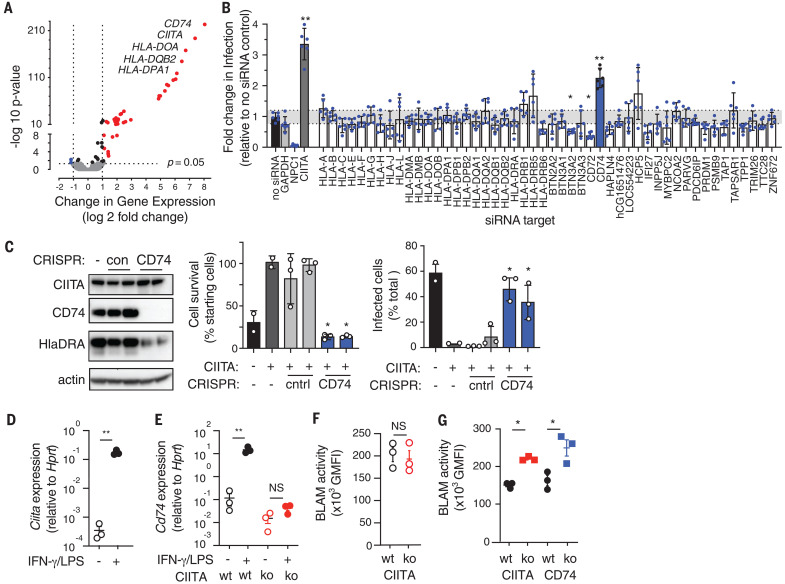
Transcriptional activity of CIITA and enhanceosome components are required for resistance. (**A**) Genes regulated by CIITA in U2OS cells, with strongest induced genes identified. Mean of three independent CIITA-expressing clones and controls. (**B**) EboGP-VSV infection of CIITA-expressing cells treated with small interfering RNA (siRNA) against CIITA transcriptional targets. Data are from two siRNAs per gene, *N* = 3 independent screens, and bars indicate means with 95% confidence intervals (CIs). One-way ANOVA with Bonferroni’s multiple comparisons; *P < 0.05; **P < 0.01. Dotted lines indicate 99% CIs from no siRNA control. (**C**) *CD74* CRISPR-targeting in CIITA-overexpressing U2OS cells was verified by immunoblot, and infection and survival were measured after EboGP-VSV challenge. Data are means ± SEM of *N =* 3 experiments using two independent cell clones. (**D** and **E**) *Ciita* and *Cd74* expression in wild-type (wt) or *Ciita*^−/−^ mouse BMDMs with or without priming by IFN-γ and LPS. ko, knockout; NS, not significant. (**F** and **G**) Fusion of EboGP-VLPs in unprimed (F) or primed (G) mouse BMDM from *Ciita*^−/−^ and *Cd74*^−/−^ mice, measured as geometric mean fluorescence (GMFI) of cleaved CCF2. BLAM, β-lactamase. Data are means ± SEM for independent cultures from three mice per group. Student’s *t* test; *P < 0.05; **P < 0.01. Similar results were observed in three independent experiments.

CIITA, also known as NLRA, is a nucleotide-binding oligomerization domain (Nod)–like receptor (NLR) (*11*), but unlike most other NLRs, which function as cytosolic sensors, CIITA is a transcription factor (*12*). We therefore hypothesized that its antiviral activity occurred through the altered expression of host target genes. Supporting this hypothesis, mutation of domains required for transcriptional activity completely ablated CIITA antiviral activity (fig. S4). Resistance also required NF-Y, a component of the enhanceosome multiprotein complex, which mediates transcriptional activation by CIITA (*13*), but resistance was independent of another enhanceosome protein, RFX5 (figs. S4 and S5). Antiviral activity was therefore mediated by a subset of NF-Y–dependent, RFX5-independent CIITA target genes, which includes genes associated with antiviral immunity (*14*). Systematic knockdown of all CIITA target genes identified a single gene, *CD74*, required for CIITA-mediated resistance (Fig. 3, A and B). This was confirmed by CRISPR knockout of CD74 expression and function in CIITA-overexpressing cells (Fig. 3C).

Both CIITA and CD74 are expressed at high levels by macrophages and dendritic cells (DCs), which are early targets of EBOV (*15*, *16*). To test whether CIITA has antiviral activity in immune cells, we used primary bone marrow–derived macrophages (BMDMs) from *Ciita^−/−^* and *Cd74^−/−^* mice. Naïve BMDMs did not express high levels of CIITA or CD74, and they showed no difference in viral fusion. Treatment with interferon-γ (IFN-γ) and lipopolysaccharide (LPS) induced expression of CIITA and CD74, and *Ciita*^−/−^ and *Cd74^−/−^* BMDMs primed with IFN-γ and LPS had higher levels of EboGP-VLP fusion than those observed in equivalent wild-type cells (Fig. 3, D to G, and fig. S6). Similar results were seen in *Cd74^−/−^* bone marrow–derived DCs and in a *CD74*^−/−^ human macrophage-like cell line (differentiated THP-1) (figs. S7 and S8). Thus, endogenous CIITA and CD74 have antiviral activity in primary immune cells, which can be induced by exposure to IFN-γ and LPS.

**Fig. 4:**
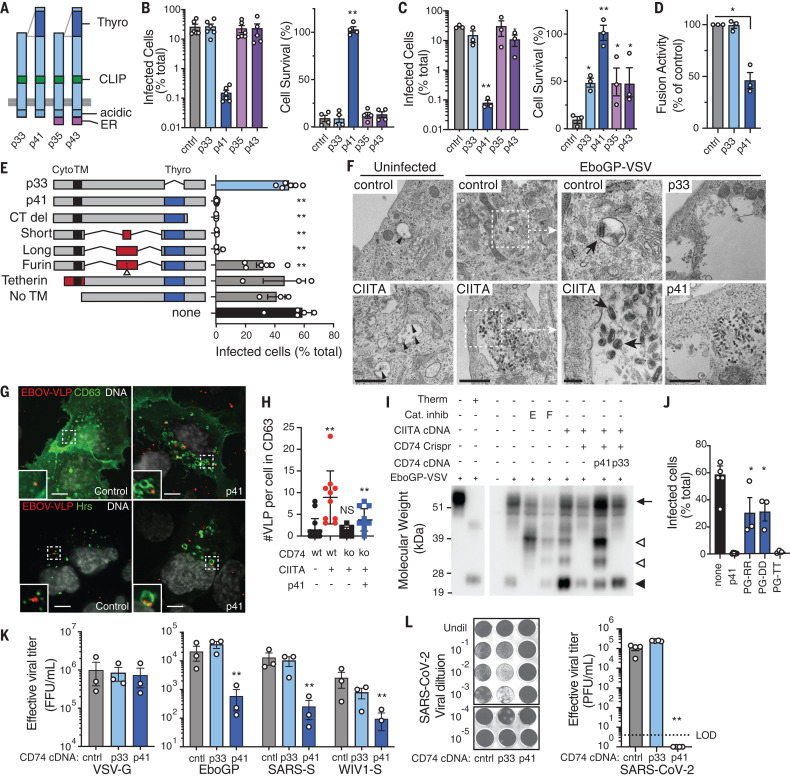
CD74 p41 inhibits cathepsin-mediated cleavage of EboGP. (**A**) Human CD74 isoforms with ER retention signal (ER), CLIP, acidic, and p41 thyroglobulin (Thyro) domains. (**B** and **C**) EboGP-VSV infection and survival of *Cd74*^−/−^ CIITA-expressing (B) or wt (C) U2OS cells expressing CD74 isoforms. (**D**) EboGP-VLP fusion in THP-1 macrophage-like cells expressing CD74 p33 and p41. (**E**) EboGP-VSV infection of U2OS cells expressing CD74 mutant constructs. Cyto, cytoplasmic domain; TM, transmembrane domain; Thyro, thyroglobulin domain; CT del, carboxy-terminus deletion; No TM, deletion of the transmembrane sequence. (**F**) Transmission electron micrographs of control, CIITA-expressing, and CD74-expressing U2OS cells 3 hours after infection with EboGP-VSV. Dotted-line regions are enlarged in adjacent panels (as indicated by white arrows). Intraluminal vesicles (black arrowheads) and internalized EboGP-VSV (black arrows) are marked. Scale bars, 1 μm (left, center left, and right panels) and 200 nm (center right panels). (**G**) Confocal microscopy of control and p41-expressing U2OS cells showing EBOV-VLP (red), CD63, or Hrs (green), and nuclei (white). Scale bars, 10 μm. (**H**) VLPs associated with CD63 endosomes in U2OS cells expressing CIITA and CD74 as indicated. Each point represents a single cell, mean ± SD *n* ≥ 9. Mann-Whitney *U* test; **P < 0.01. Similar results were seen in three independent experiments. (**I**) Immunoblot of EboGP in EboGP-VSV–infected U2OS cells. EboGP-VSV preparation ± thermolysin (Therm) is shown for reference (left). Cells were treated with cathepsin inhibitors (Cat. inhib) E64D (E) or FYDMK (F), or expressed CIITA and CD74. EboGP in virus particles (arrow), after proteolysis (closed arrowhead), and after partial cleavage (open arrowhead) are indicated. (**J**) EboGP-VSV infection of U2OS cells expressing p41 with CTSL binding site mutations. (**K**) Infection of control, p33-, or p41-expressing U2OS cells by HIV-GFP pseudotyped with GPs from VSV, EBOV, SARS-CoV, or WIV1-CoV, measured as focus-forming units per milliliter of virus (FFU/ml). (**L**) Infection of control, p33-, or p41-expressing Vero cells by SARS-CoV-2, showing representative crystal violet-stained monolayers and infection measured as plaque-forming units per milliliter of virus (PFU/ml). Except where indicated, data are means ± SEM of data from ≥3 independent experiments. Student’s *t* test with Benjamini correction; *P < 0.05; **P < 0.01.

CD74 is the major histocompatibility complex class II (MHC-II) invariant chain, and human cells express four main isoforms of CD74, which differ in the presence of an N-terminal endoplasmic reticulum (ER) retention signal and an internal thyroglobulin domain (Fig. 4A) (*17*). Only one CD74 isoform, p41, was able to fully rescue resistance to EboGP-VSV infection in CIITA-expressing, CD74-knockout cells (Fig. 4B and fig. S9). p41 conferred resistance independently of CIITA expression (Fig. 4C), which demonstrates that CD74 p41 expression was sufficient to induce antiviral activity. This property of CD74 was not limited to U2OS cells, as CD74 p41 similarly inhibited fusion when expressed in THP-1 cells (Fig. 4D). The p41 isoform contains the thyroglobulin domain, lacks the ER retention signal, and normally accumulates in endosomes. Mutant constructs of CD74 revealed that only the thyroglobulin domain is essential for antiviral activity, but dissociation from the membrane—either by addition of a furin cleavage site (labeled furin in Fig. 4E) or deletion of the transmembrane sequence (No TM in Fig. 4E)—or delivery to the cell surface by fusion to a heterologous cytoplasmic and transmembrane sequence from tetherin (tetherin in Fig. 4E) almost completely removed antiviral activity (Fig. 4E and fig. S10). Thus, antiviral activity required delivery of the thyroglobulin domain to the endosomal membrane. Electron microscopy showed that EboGP-VSV virions accumulated in late endosomal multivesicular bodies (MVBs) of CIITA- and CD74 p41–expressing cells, with some virions within intraluminal vesicles (Fig. 4F and fig. S11). Confocal microscopy confirmed that virus-like particles (VLPs) localized proximal to CD63 and the ESCRT component Hrs, which mark MVBs (*18*, *19*) (Fig. 4, G and H). Thus, CIITA and CD74 p41 inhibit fusion by arresting viral particles in MVB compartments.

EBOV entry requires endosomal cathepsins (*4*, *10*, *20*) (fig. S12), which sequentially process EboGP (Fig. 4I and fig. S13). The CD74 thyroglobulin domain inhibits cathepsins (*21*), which suggests that this may be the mechanism for antiviral activity. In support of this, CD74 inhibited EboGP processing, similar to the effects of the cathepsin L (CTSL) inhibitor FYDMK (Fig. 4I). Additionally, disruption of the p41 CTSL binding site (*22*, *23*) by mutation completely inhibited antiviral activity (Fig. 4J and fig. S10). GP cleavage by endosomal proteases facilitates the entry of other viruses, including coronaviruses. SARS-CoV and SARS-CoV-2 S proteins can be processed by either endosomal cathepsin B and CTSL or alternatively by cell-surface serine proteases including TMPRSS2 (*24*, *25*). In TMPRSS-expressing cells, such as lung epithelium, inhibition of both cathepsins and serine proteases is required to inhibit viral entry, whereas cathepsin inhibitors alone block infection in cell lines—such as U2OS and Vero cells—that lack TMPRSS2 (*25*). p41 inhibited the entry of viruses pseudotyped with S proteins from SARS-CoV and a related bat virus, WIV1-CoV, into U2OS cells, which demonstrates that p41 inhibited S protein processing (Fig. 4K). To determine whether p41 exhibited antiviral activity against authentic SARS coronavirus, we challenged p41-expressing Vero E6 cells with SARS-CoV-2. CD74 p41 expression completely inhibited plaque formation, which demonstrates that this antiviral activity extended beyond filoviruses (Fig. 4L).

Here, we identify the antiviral activity of CIITA and CD74. We show that CIITA induces resistance by up-regulation of the p41 isoform of CD74, which blocks cathepsin-mediated cleavage of viral GPs, thereby preventing viral fusion. This antiviral activity protects against a wide range of cathepsin-dependent viruses, including filoviruses and coronaviruses; functions in macrophages and DCs that are early targets of infection (*15*, *16*); and is activated by IFN-γ. We demonstrate that CIITA and CD74 mediate the endosomal sequestration of certain viruses as a mechanism of cellular host defense. We speculate that this activity is evolutionarily ancient and precedes their better-known role in antigen processing. We anticipate that the application of this transposon screening approach to other models of infection will reveal additional mechanisms that have eluded conventional screening strategies.

## Supplementary Materials

science.sciencemag.org/content/370/6513/241/suppl/DC1 Materials and Methods Figs. S1 to S14 Tables S1 to S5 References (*26*–*43*)

## Appendix

View/request a protocol for this paper from *Bio-protocol*.
